# Supplementary material for: In Vitro Biological Evaluation of Aprepitant Based 177Lu-Radioconjugates
Source: Pharmaceutics. 2022 Mar 10;14(3):607. doi: 10.3390/pharmaceutics14030607 (PMC8949964; doi:10.3390/pharmaceutics14030607)
Supplement: Supplementary file 1 [file pharmaceutics-14-00607-s001.zip › pharmaceutics-1611282 - Supplementary materials.pdf]

Article

In Vitro Biological Evaluation of Aprepitant Based ^{177}Lu-Radioconjugates

Paweł K. Halik [1,*], Przemysław Koźmiński [1], Joanna Matalińska [2], Piotr F. J. Lipiński [2], Aleksandra Misicka [2,3] and Ewa Gniazdowska [1]

[1] Centre of Radiochemistry and Nuclear Chemistry, Institute of Nuclear Chemistry and Technology,
03-195 Warsaw, Poland; p.kozminski@ichtj.waw.pl (P.K.);
e.gniazdowska@ichtj.waw.pl (E.G.)

[2] Department of Neuropeptides, Mossakowski Medical Research Institute, Polish Academy of Sciences,
02-106 Warsaw, Poland; jmatalinska@imdik.pan.pl (J.M.); plipinski@imdik.pan.pl (P.F.J.L.);
misicka@chem.uw.edu.pl (A.M.)

[3] Faculty of Chemistry, University of Warsaw, 02-093 Warsaw, Poland

* Correspondence: p.halik@ichtj.waw.pl; Tel.: +48-22-504-13-16

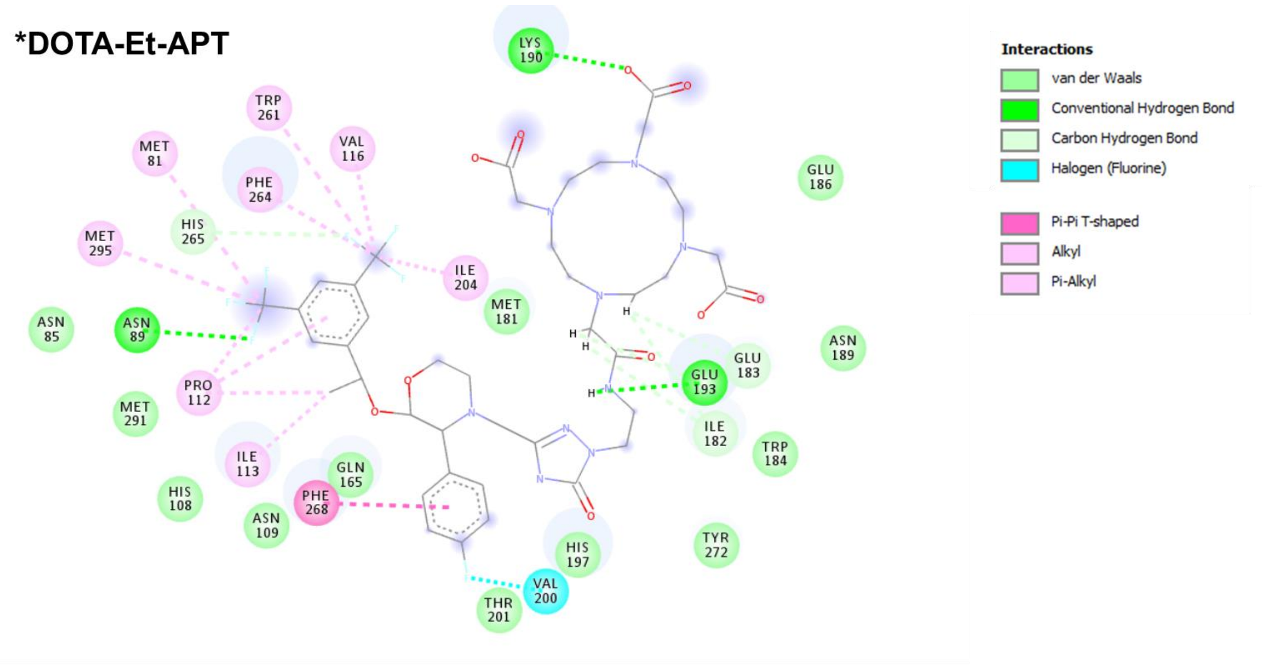

Figure S1. Scheme of the interactions of ***DOTA-Et-APT** with the NK1R. *Interaction types are shown in the legend (top right corner).*

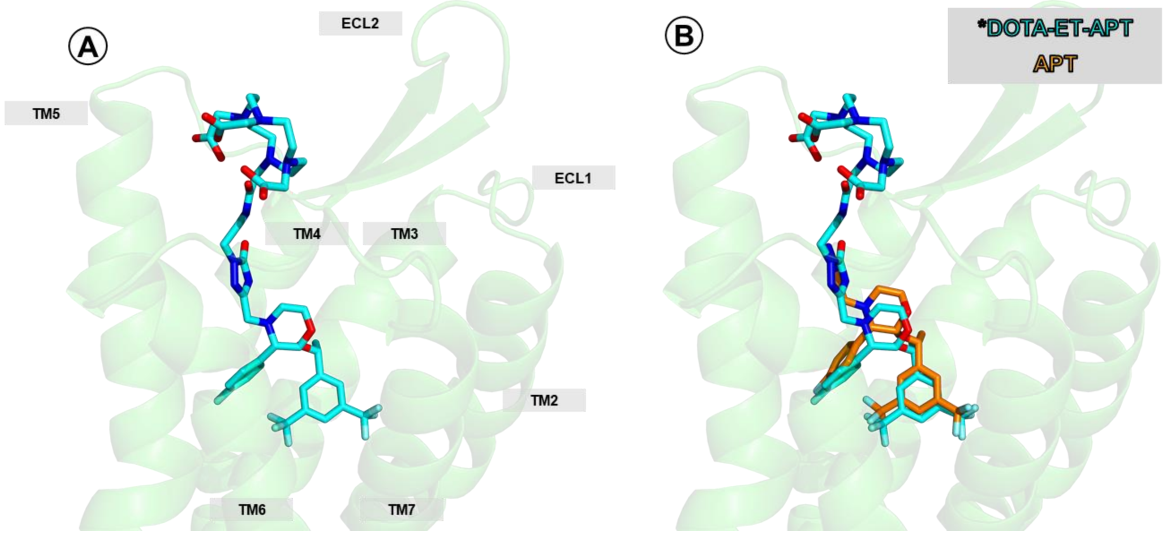

Figure S2. Binding mode of ***DOTA-Et-APT** in the NK1R (general view). **A)** the ligand alone or **B)** superposed on the crystallographic position of aprepitant (APT). ****DOTA-Et-APT*** *shown as cyan sticks. APT shown as orange sticks. Hydrogen display is suppressed. The receptor is shown in a simplified manner as green helices. TM1 not shown.*

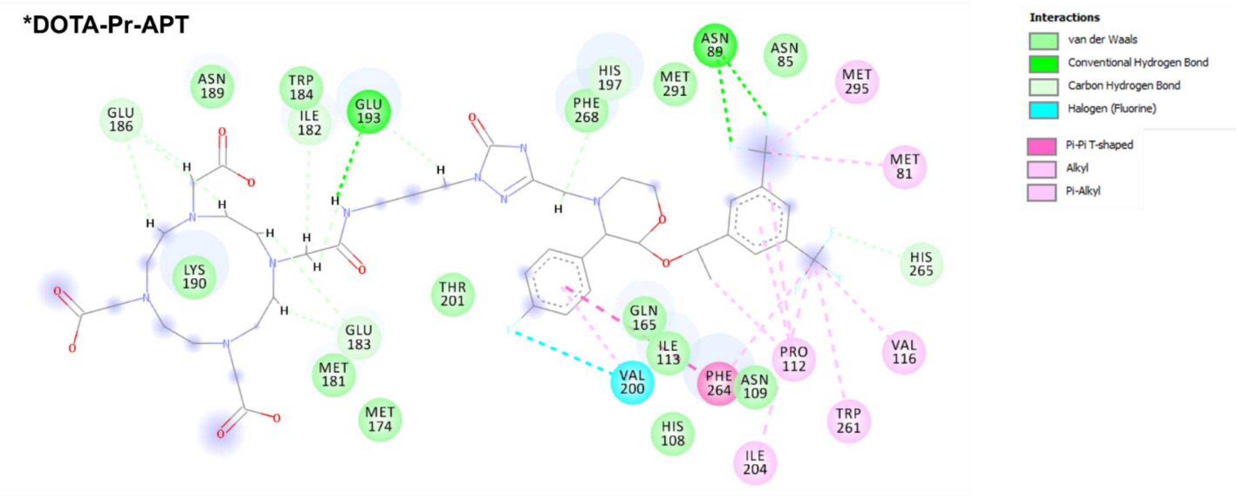

Figure S3. Scheme of the interactions of ***DOTA-Pr-APT** with the NK1R. *Interaction types are shown in the legend (top right corner).*

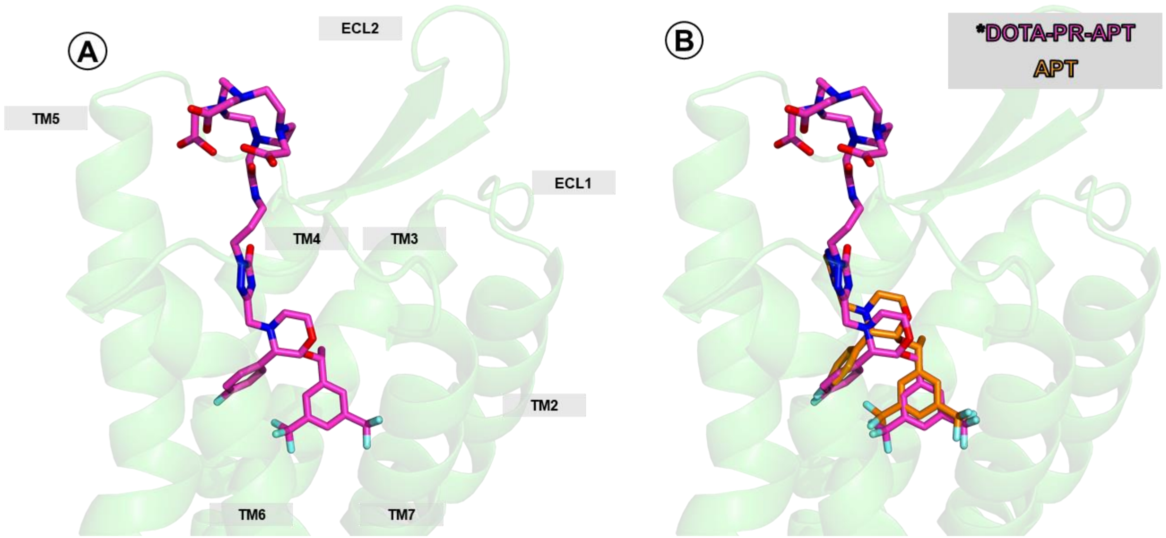

Figure S4. Binding mode of ***DOTA-Pr-APT** in the NK1R (general view). **A)** the ligand alone or **B)** superposed on the crystallographic position of aprepitant (APT). *DOTA-Pr-APT* *shown as magenta sticks. APT shown as orange sticks. Hydrogen display is suppressed. The receptor is shown in a simplified manner as green helices. TM1 not shown.*

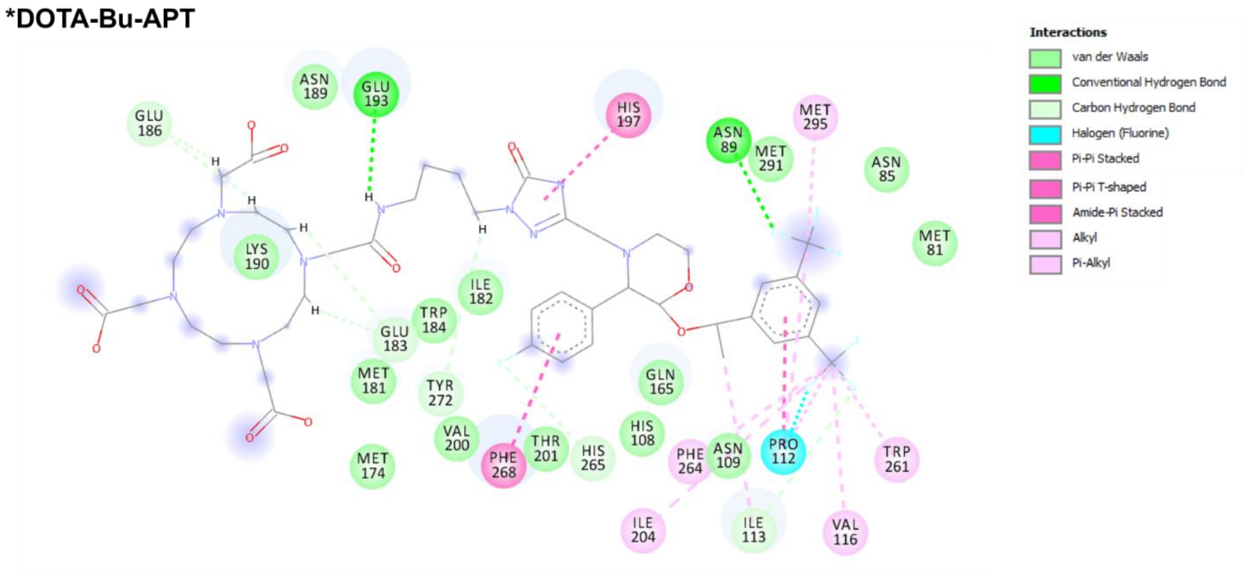

Figure S5. Scheme of the interactions of ***DOTA-Bu-APT** with the NK1R. *Interaction types are shown in the legend (top right corner).*

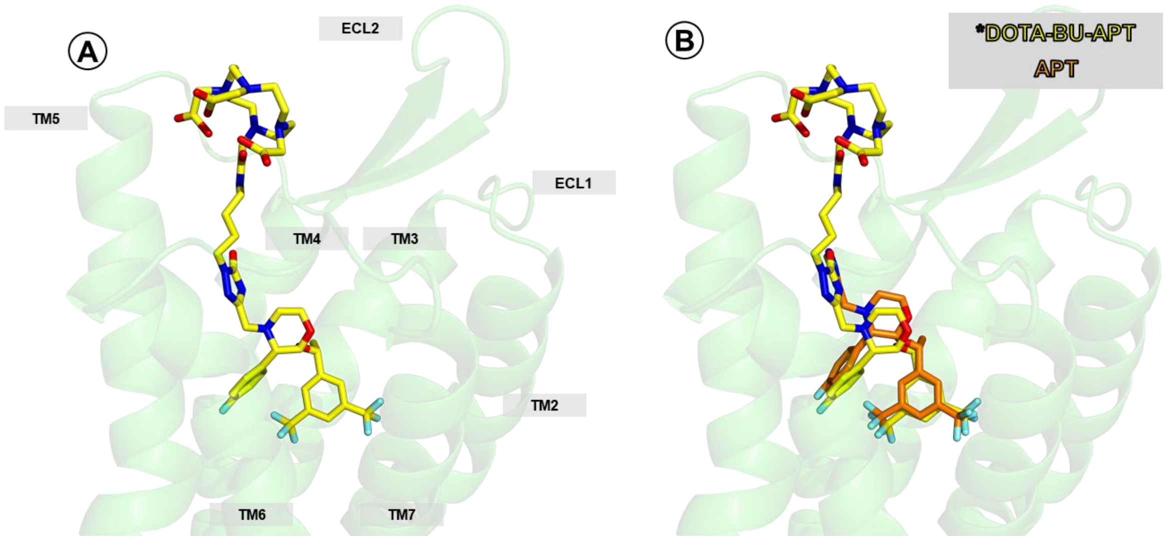

Figure S6. Binding mode of ***DOTA-Bu-APT** in the NK1R (general view). **A)** the ligand alone or **B)** superposed on the crystallographic position of aprepitant (APT). **DOTA-Bu-APT shown as yellow sticks. APT shown as orange sticks. Hydrogen display is suppressed. The receptor is shown in a simplified manner as green helices. TM1 not shown.*

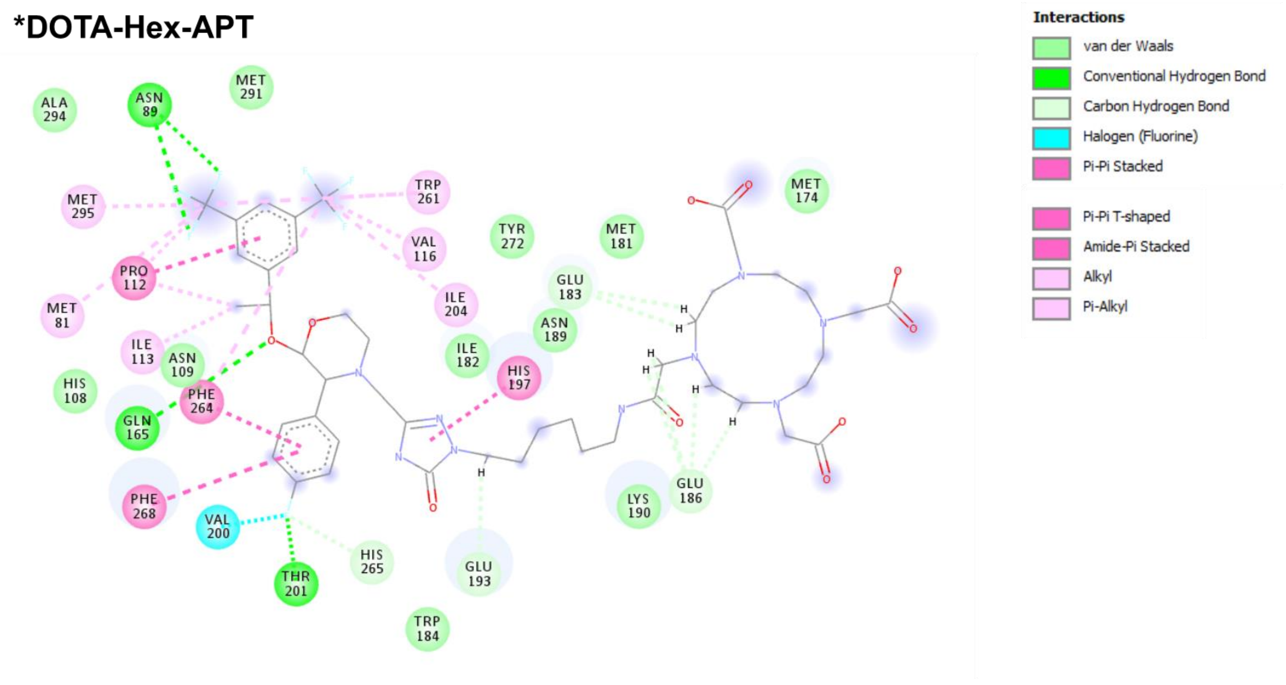

Figure S7. Scheme of the interactions of ***DOTA-Hex-APT** with the NK1R. *Interaction types are shown in the legend (top right corner).*

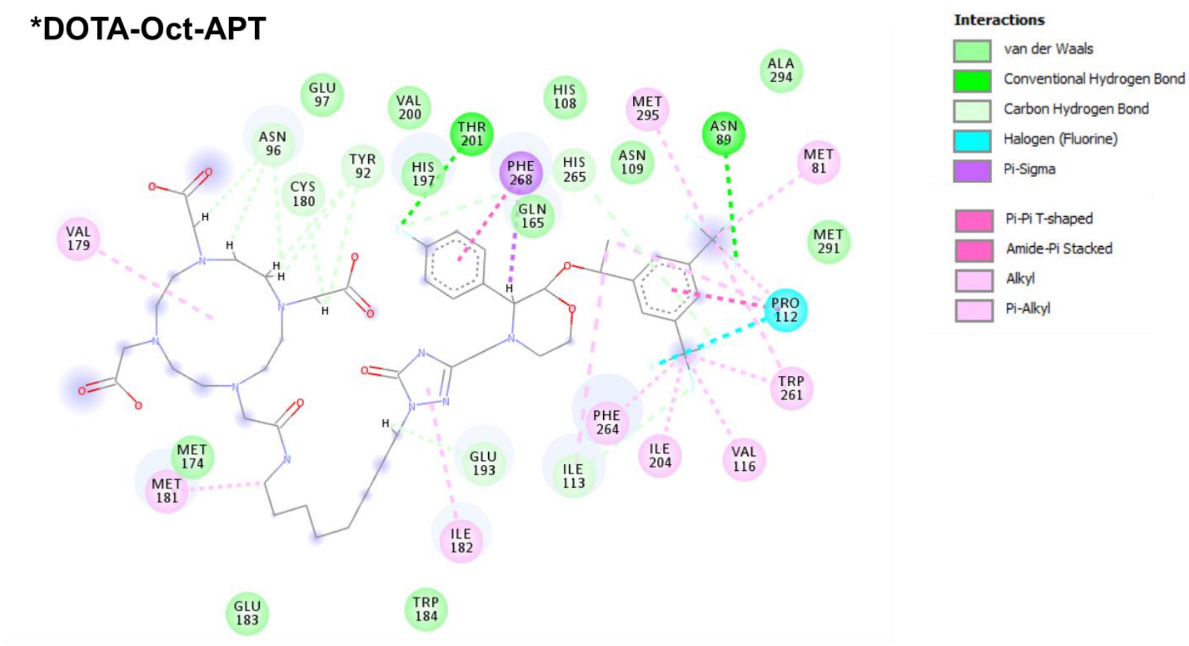

Figure S8. Scheme of the interactions of ***DOTA-Oct-APT** with the NK1R. *Interaction types are shown in the legend (top right corner).*

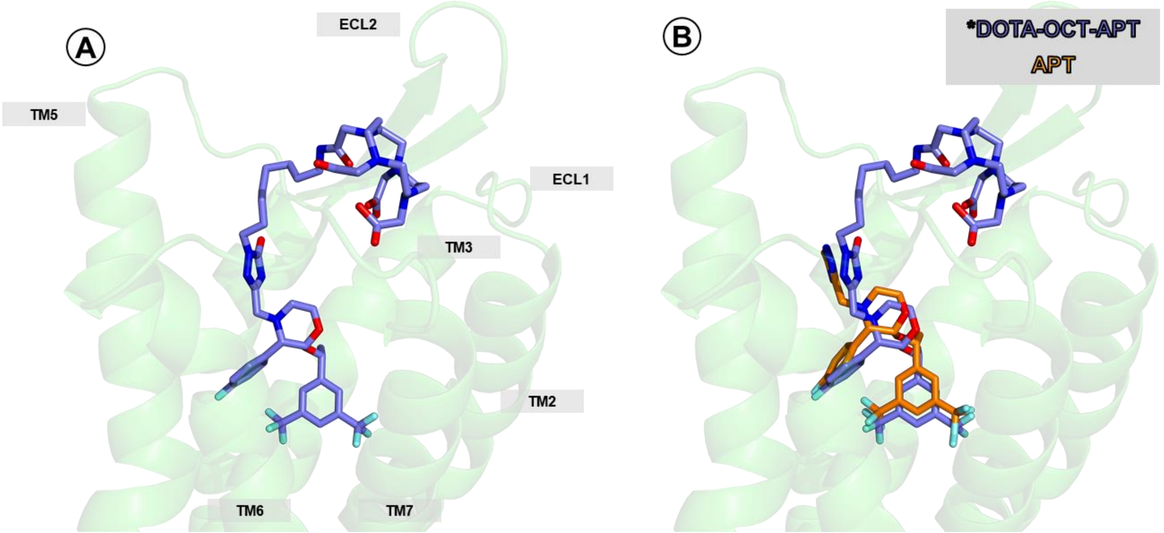

Figure S9. Binding mode of ***DOTA-Oct-APT** in the NK1R (general view). **A)** the ligand alone or **B)** superposed on the crystallographic position of aprepitant (APT). *__*DOTA-Oct-APT__ shown as slate blue sticks. APT shown as orange sticks. Hydrogen display is suppressed. The receptor is shown in a simplified manner as green helices. TM1 not shown.*

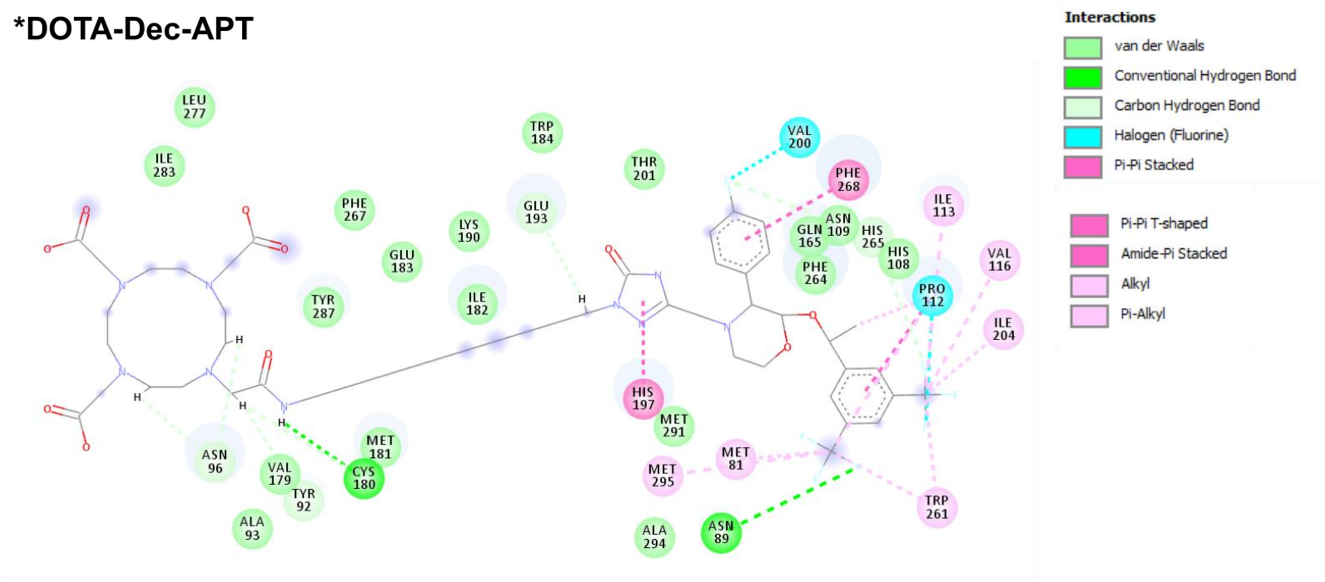

Figure S10. Scheme of the interactions of ***DOTA-Dec-APT** with the NK1R. *Interaction types are shown in the legend (top right corner).*

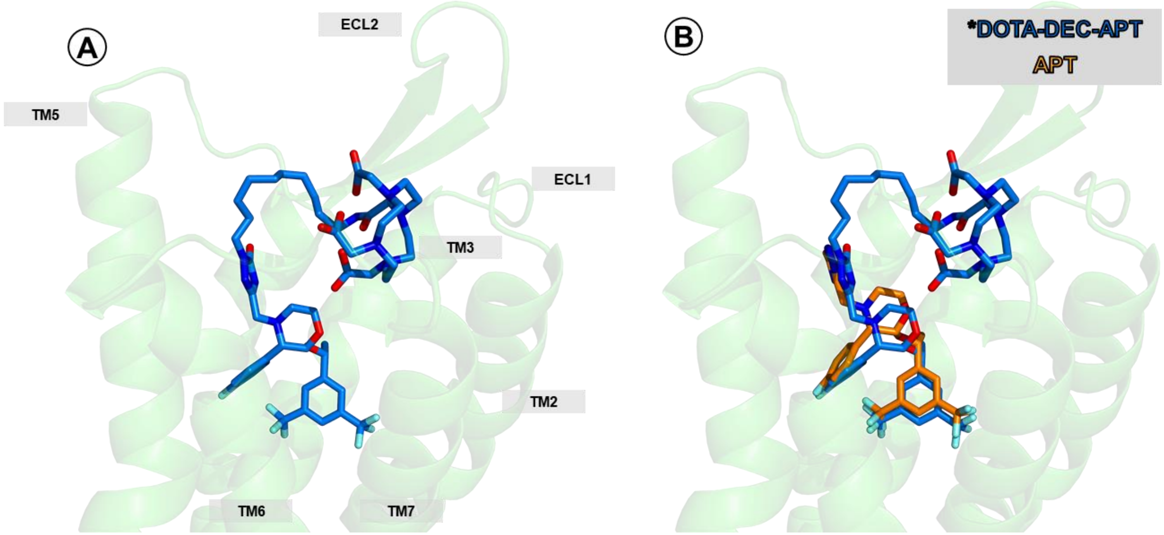

Figure S11. Binding mode of ***DOTA-Dec-APT** in the NK1R (general view). **A)** the ligand alone or **B)** superposed on the crystallographic position of aprepitant (APT). ****DOTA-Dec-APT*** *shown as marine-blue sticks. APT shown as orange sticks. Hydrogen display is suppressed. The receptor is shown in a simplified manner as green helices. TM1 not shown.*
